# Root defense analysis against *Fusarium oxysporum* reveals new regulators to confer resistance

**DOI:** 10.1038/srep05584

**Published:** 2014-07-07

**Authors:** Yi Chung Chen, Chin Lin Wong, Frederico Muzzi, Ido Vlaardingerbroek, Brendan N. Kidd, Peer M. Schenk

**Affiliations:** 1 Plant-Microbe Interactions Laboratory, School of Agriculture and Food Sciences, The University of Queensland, Brisbane, Queensland 4072, Australia

## Abstract

*Fusarium oxysporum* is a root-infecting fungal pathogen that causes wilt disease on a broad range of plant species, including *Arabidopsis thaliana*. Investigation of the defense response against this pathogen had primarily been conducted using leaf tissue and little was known about the root defense response. In this study, we profiled the expression of root genes after infection with *F. oxysporum* by microarray analysis. In contrast to the leaf response, root tissue did not show a strong induction of defense-associated gene expression and instead showed a greater proportion of repressed genes. Screening insertion mutants from differentially expressed genes in the microarray uncovered a role for the transcription factor *ETHYLENE RESPONSE FACTOR72* (*ERF72*) in susceptibility to *F. oxysporum*. Due to the role of *ERF72* in suppressing programmed cell death and detoxifying reactive oxygen species (ROS), we examined the *pub22/pub23/pub24* U-box type E3 ubiquitin ligase triple mutant which is known to possess enhanced ROS production in response to pathogen challenge. We found that the *pub22/23/24* mutant is more resistant to *F. oxysporum* infection, suggesting that a heightened innate immune response provides protection against *F. oxysporum*. We conclude that root-mediated defenses against soil-borne pathogens can be provided at multiple levels.

Plant roots are surrounded by a diverse range of microorganisms in the rhizosphere. Root-microbe interactions can be either beneficial or detrimental^1^ and a fast and accurate assessment of the surrounding organisms is essential for the plant's survival. Bacterial flagellin and other microbe-associated molecular patterns (MAMPs), act as signals for the plant to determine potential threats^2^. Plants are able to recognize MAMPs through pattern recognition receptors that specifically bind to their target MAMP, and recognition leads to activation of the plant's basal immune response^3^,^4^. MAMP detection leads to a signal transduction and amplification kinase cascade that triggers the activation of pathogenesis related (PR) proteins^5^ the production of reactive oxygen species (ROS)^6^ and many secondary metabolites, including the deposition of callose, which act as a physical and chemical barrier to prevent pathogen attack^7^.

Pathogen invasion may also lead to the activation of further hormone-controlled defense pathways, such as systemic acquired resistance (SAR) which protects against subsequent infections^8^. SAR is mediated by salicylic acid (SA) signaling but has also been shown to require jasmonate (JA) in the initial stages^9^. The SA- and JA/ethylene- signaling pathways are generally considered to be effective against biotrophic and necrotrophic pathogens, respectively^10^,^11^. Hemi-biotrophic pathogens, such as *Phytophtora infestans*, typically start out as a biotrophic pathogen, however later in the infection cycle the pathogen changes to a necrotrophic lifestyle that is often accompanied by the production of cellulolytic enzymes and toxins to damage host cells enabling further invasion and nutrient uptake. This change in lifestyle requires hemi-biotrophic pathogens to be adept at hijacking host signaling pathways.

*Fusarium oxysporum* is a root-infecting pathogen that infects a number of plants, including cotton, tomato, banana and *Arabidopsis*. *F. oxysporum* acts as a hemi-biotrophic pathogen in *Arabidopsis* and the application of SA on *Arabidopsis* leaves resulted in a partial increase in resistance^12^. Mutants deficient in SA-mediated defense were shown to be more susceptible to *F. oxysporum*. For instance, the *sid2* mutant is impaired in SA biosynthesis and is susceptible to *F. oxysporum* f. sp. *conglutinans*^13^,^14^. However, during infection *F. oxysporum* strongly induces JA-mediated defense responses in the leaves^15^.

Interestingly, there is evidence that plants show increased resistance to *F. oxysporum* when they are insensitive to jasmonic acid (JA). The *jasmonate insensitive1 (jin1*) mutant, otherwise known as *myc2*, shows increased resistance to *F. oxysporum*^16^. Similarly, the *coi1* (*coronatine insensitive1*) and *pft1* (*phytochrome and flowering time1*) mutants that are also compromised in JA responses were later shown to possess increased resistance to *F. oxysporum*^17^,^18^,^19^. Therefore, modulation of plant defense signaling can potentially be used to increase plant defense to *F. oxysporum*. However, recently-provided evidence show that not all *formae speciales* of *F. oxysporum* utilize the jasmonate pathway to promote infection in Arabidopsis^20^. Therefore different *formae speciales* may adopt different strategies to cause disease in the host.

To counteract the pathogen's attempt to cause disease, plants possess Resistance (*R*) gene loci to recognize the pathogen and enable an efficient defense response. In *Arabidopsis* seven *R* genes have been identified, termed *RESISTANCE TO FUSARIUM* (*RFO1-RFO7*), using a cross between resistant wild-type (WT) Columbia-0 (Col-0) and the more susceptible Taynuilt-0 (Ty-0) ecotype^14^,^21^,^22^. *RFO1* was found to encode a wall-associated kinase-like kinase 22 (WAKL22) and *RFO2* encodes a receptor-like protein^14^,^22^. The *RFO* genes are unique in that they provide protection to multiple *formae speciales* of *F. oxysporum*.

Expression of the resistant Col-0 alleles of *RFO1* and *RFO2* in the Ty-0 ecotype led to a restriction of pathogen growth in the roots^22^. In addition grafting of the resistant *coi1* mutant with the WT Col-0 showed that the root tissue of *coi1* was responsible for the resistance of this mutant^18^. The *coi1* mutant also shows restriction of fungal growth in the roots^20^. Despite these findings, there is little known about root defense responses against *F. oxysporum*, particularly after infection of *Arabidopsis* roots. The present study aimed to investigate transcriptional responses to *F. oxysporum* in *Arabidopsis* roots. Genome-wide microarray data obtained 48 h after inoculations were used as a basis to evaluate *F. oxysporum* infection. The results from this analysis revealed only very weak gene induction in the roots and most differentially expressed genes showed reduced expression during *F. oxysporum* infection. An insertion mutant in one of the suppressed genes *ETHYLENE RESPONSE FACTOR72* (*ERF72*) showed increased resistance. *ERF72* has previously been shown to have a role in suppressing programmed cell death^23^. We investigated the cause of resistance in the *erf72* line and looked at the role of early plant MAMP defense responses against *F. oxysporum* infection.

## Results

### Infection of *Arabidopsis* with *Fusarium oxysporum* circumvents plant defence and leads to repression of many root genes

To identify root genes that play a role in the interaction of *F. oxysporum* with *Arabidopsis thaliana* Col-0 plants, we performed three independent microarray experiments using *F. oxysporum*-infected root tissue harvested at 48 h after infection. We chose to analyse gene expression at 48 hours after infection to compare with a previously published microarray conducted on the leaf tissue of *F. oxysporum*-infected *Arabidopsis*^15^,^17^. Overall, we found 89 genes that were significantly differentially regulated greater than 1.5-fold (*p* < 0.05). Of these genes, the majority (72 genes) were found to be repressed by more than 1.5-fold in the infected root tissue relative to the mock-inoculated roots, whereas only 17 genes were found to be induced more than 1.5-fold by *F. oxysporum* infection (Table 1; Table 2; Supplementary Table 1). Of the significantly induced genes, only two were induced greater than two-fold, compared to 15 genes that were repressed greater than two-fold (Table 1 and Table 2). Therefore, at the 48 h time point tested, this microarray experiment suggests that *F. oxysporum* primarily repressed genes in the roots of *Arabidopsis*.

To independently confirm the results of microarray analyses, we performed quantitative real-time reverse transcriptase PCR (qRT-PCR) on *F. oxysporum*-infected plants in three separate biological experiments at 48 h post inoculation. Results from qRT-PCR confirmed the differential expression from the microarray data for all genes that were tested (Supplementary Figure 1). We next compared our microarray results with the microarray analyses previously performed on the leaves of *F. oxysporum*-infected *Arabidopsis* plants^15^,^17^. The inoculation method was the same for the root microarray, but only leaf tissues were collected. Interestingly, of the total number of genes significantly induced or repressed in both studies, only three genes were common to both microarray experiments. These genes were At1g60590 which encodes a pectin lyase-like protein, At2g47400 which encodes a CP12 protein that forms a complex with glyceraldehyde 3-phosphate dehydrogenase and At5g25980 which encodes the myrosinase TGG2 involved in glucosinolate metabolism. All three genes were suppressed in both microarray experiments (Table 1)^15^. Furthermore, we compared our microarray with the microarray of Iven et al^24^, which examined root gene expression changes in response to *Verticillium longisporum* infection in *Arabidopsis*. At2g47400 (CP12) was also differentially regulated in this microarray suggesting that the CP12 protein may be involved in resistance against root pathogens. We inoculated a T-DNA mutant of the CP12 gene with *F. oxysporum*, however the mutant did not show any significant change in disease symptoms after infection (Supplementary Figure 2).

We also compared our root microarray data to the RNAseq experiment of Zhu et al.^25^. The authors of this study infected two weeks-old seedlings grown with *F. oxysporum* and analysed gene expression at 1 and 6 days post inoculation. However none of the differentially expressed genes from our microarray were found in the differentially expressed genes from this study. However, Zhu et al.^25^ found similar patterns of gene expression with our previously carried out shoot microarray, such as the up-regulation of JA-responsive genes and genes involved in the tryptophan and glucosinolate pathway^15^. As the tissue used by Zhu et al.^25^ contained whole plants it is possible that gene expression from the shoot material masked the differential expression of root specific genes in their analysis.

Overall, we found that gene expression in the *F. oxysporum*-infected roots was very distinct when compared to leaves. We found the majority of the differentially expressed genes in the root microarray to be repressed in response to *F. oxysporum* infection whereas in the leaf the majority of differently expressed genes were induced upon *F. oxysporum* infection and therefore this suggests a major functional difference in plant defence that is activated between roots and shoots upon *F. oxysporum* infection.

In the leaf microarray, the highest differentially expressed genes were the related *PLANT DEFENSIN1.2* genes, *PDF1.2a* and *PDF1.2b*, along with *PATHOGENESIS RELATED4* (*PR4*) which encodes a hevein-like protein^15^,^17^. These defense genes were induced quite strongly in the leaves (up to 40-fold for *PDF1.2a*) and are considered marker genes for the jasmonate-associated defense response. Accordingly, a number of other JA-associated genes were also up-regulated in the leaf microarray experiment^15^. In our root microarray experiment, the strongest inducible gene encoded an oxidoreductase known as *JASMONATE REGULATED GENE 21* which was only induced approximately 2-fold (Table 2). We also found the *JAZ8* gene, encoding the JASMONATE ZIM DOMAIN8 protein which acts as a repressor of JA-associated transcription factors, to be induced. However we could not find strong induction of pathogenesis- or defense-related genes in the root microarray. When we looked at the significantly expressed genes that were below the 1.5-fold cut-off we found the JAZ1 repressor (At1g19180) as well as a plant defensin family member (At4g22214). Therefore, while some JA-related genes appear to be induced in the roots, pathogenesis-related proteins were generally not highly expressed in the root in response to *F. oxysporum* infection.

**Figure 1:**
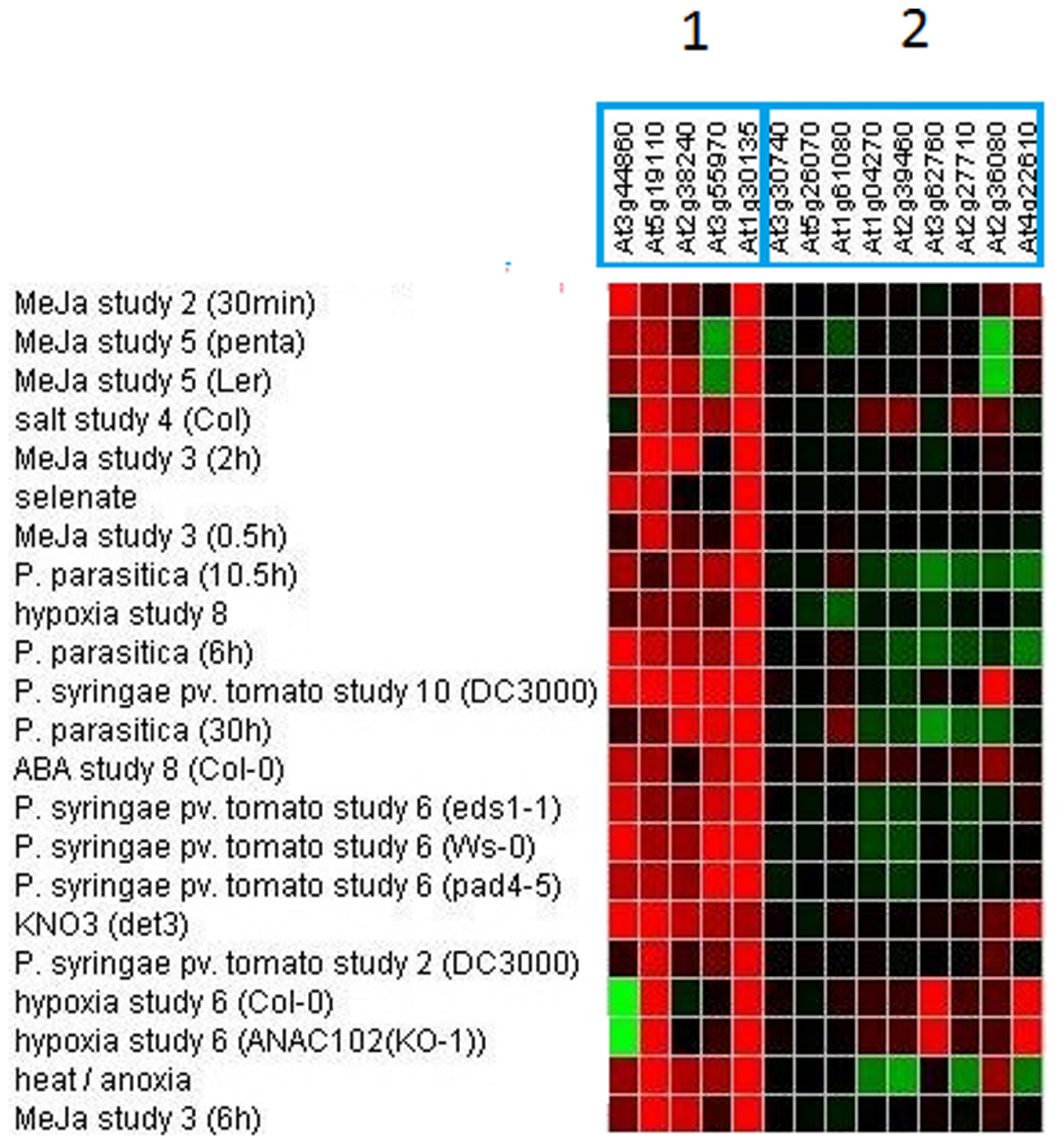
Co-regulation of *F. oxysporum*-induced *Arabidopsis* root genes. Shown is a heat map with different intensity *Arabidopsis* gene expression of various other treatments for the genes that were identified in this study to be induced in *F. oxysporum*-infected roots. Red = induced, green = repressed gene expression; data were extracted from Genevestigator^26^.

**Figure 2:**
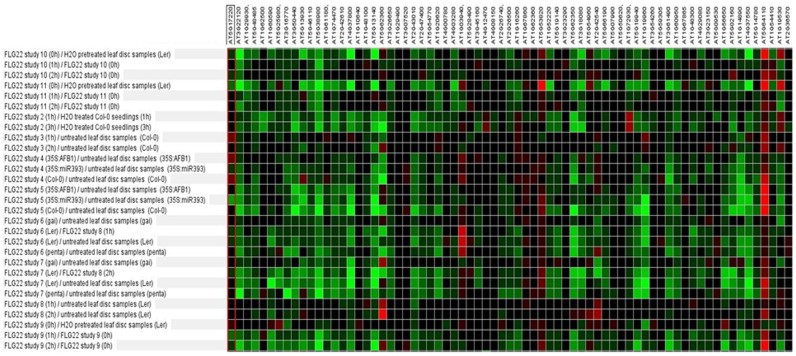
Co-regulation of *F. oxysporum*-repressed *Arabidopsis* root genes. Shown is a heat map with different intensity gene expression of FLG22-treated *Arabidopsis* plants for the genes that were identified in this study to be repressed in *F. oxysporum*-infected roots. Red = induced, green = repressed gene expression; data were extracted from Genevestigator^26^.

The relative absence of defense gene activation in the root array was surprising and we investigated further the types of genes that were induced by comparing our microarray gene lists with publically available microarray data to determine what other stimuli might affect our induced genes (26; Figures 1–2). We analysed the 17 up-regulated genes and found the genes clustered into two different groups based on their expression pattern: the first cluster of genes (At3g44860, At5g19110, At2g38240, At3g55970, At1g30135) were induced by methyl jasmonate (MeJA), *Pseudomonas syringae* inoculation and/or other abiotic treatments such as salt and heat treatment (Figure 1). The second cluster of genes (At1g04270, At2g27710, At2g39460, At2g36080, At3g30740, At5g26070) did not seem to respond highly to any treatment (Figure 1). Four of these six genes are ribosomal proteins which would explain their low responsiveness to treatments in other array experiments. It is unknown why these proteins showed differential expression in our root array. We next investigated the genes down-regulated by *F. oxysporum* with the publically available microarray data. Although the down-regulated genes were affected by a range of treatments, clustering by treatment showed that the majority of the *F. oxysporum* down-regulated genes were repressed in response to flagellin as well as treatment with *P. syringae*, and syringolin (Figure 2). Similarly to *F. oxysporum, P. syringae* is considered a hemi-biotroph and both pathogens hijack the JA pathway to promote disease susceptibility in the plant^18^,^27^,^28^,^29^. Therefore it is interesting that the genes that were induced and repressed in the *F. oxysporum*-infected root microarray were also induced and repressed in response to *P. syringae* infection. In addition, as the genes that were repressed by *F. oxysporum* were also suppressed in response to FLG22 treatment (Figure 2), this suggests that genes that are switched off during the response to FLG22 may also be suppressed in the roots during *F. oxysporum* infection. However we did not find co-expression of FLG22-induced genes when comparing the *F. oxysporum*-induced genes in Genevestigator^26^.

### An *erf72* knock-out line shows resistance to *F. oxysporum*

**Figure 3:**
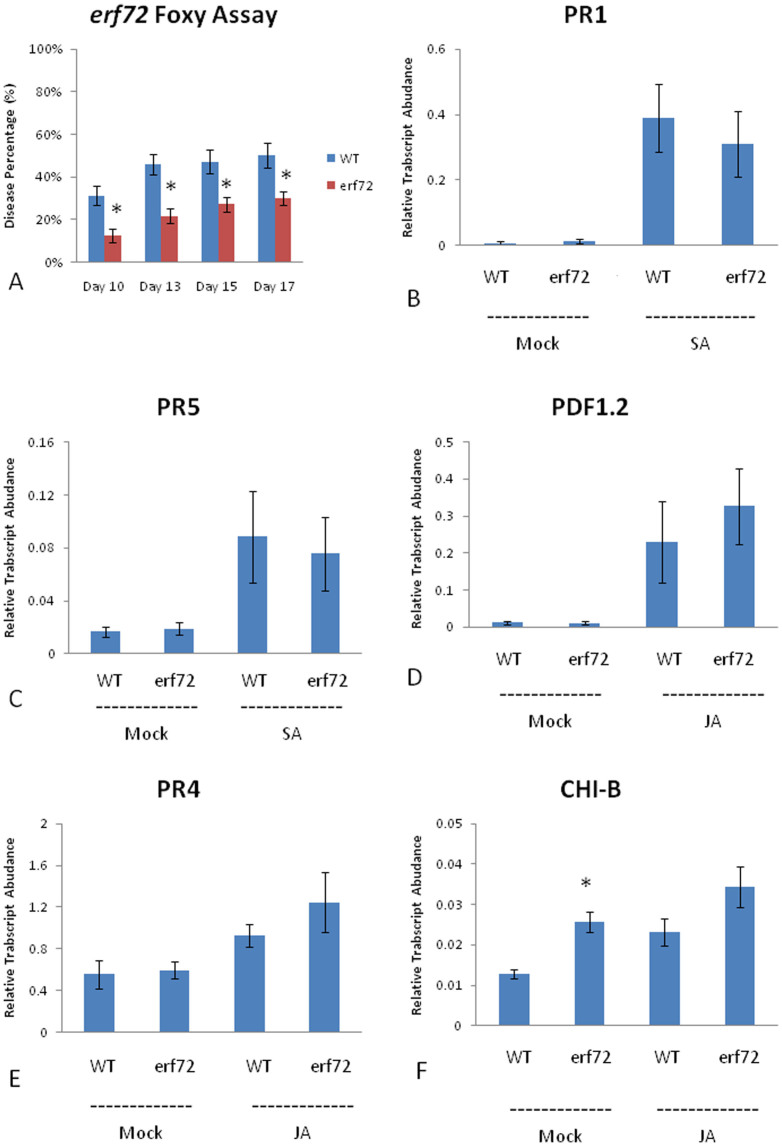
Disease scores for *erf72* mutants and different relative gene transcript abundances for WT/*erf72* plants with JA or SA treatments. The disease scores represent the average proportion of symptomatic leaves per total leaves per plant. An asterisk (*) indicates a *p*-value < 0.05; bars represent mean values ±SE of three independent biological replicates containing 10 pooled plants each (or 30 pooled plants each for qRT-PCR data).

To test whether the genes identified from our expression study play a role in defense against *F. oxysporum*, we obtained T-DNA insertion mutants for five differentially expressed genes (AT3G55970, AT4G22610, AT1G62500, AT3G16770, AT3G62670) and performed disease resistance assays with *F. oxysporum*. One of the mutants tested, *erf72*, which contains a T-DNA insertion in the *AP2/ETHYLENE RESPONSE FACTOR72* gene, showed increased resistance to *F. oxysporum* (Figure 3), suggesting that ERF72 is a negative regulator of plant defense against *F. oxysporum*. To examine how the *erf72* mutant may be providing resistance to *F. oxysporum*, we looked at the expression of a number of JA- and SA-associated marker genes after treatment with either MeJA or SA, respectively. Quantitative RT-PCR experiments showed no significant change in the expression of SA-associated *PATHOGENESIS RELATED* genes; *PR1* and *PR5*, or the JA-associated defense genes *PDF1.2* and *PR4* (Figure 3). However, the expression of the *BASIC CHITINASE* (*CHI-B*) gene, otherwise known as *PR3*, showed increased expression in the *erf72* mutant compared to WT under mock conditions (Figure 3). The heightened expression of *CHI-B* could potentially explain the increased resistance of the *erf72* mutant to *F. oxysporum* by degrading fungal hyphae in the roots and limiting infection. We therefore examined *F. oxysporum* growth within the roots of WT and *erf72* mutants using a β-glucoronidase (GUS)-expressing strain of *F. oxysporum*. However, no difference in root colonization could be identified after GUS staining (Supplementary Figure 3).

**Figure 4:**
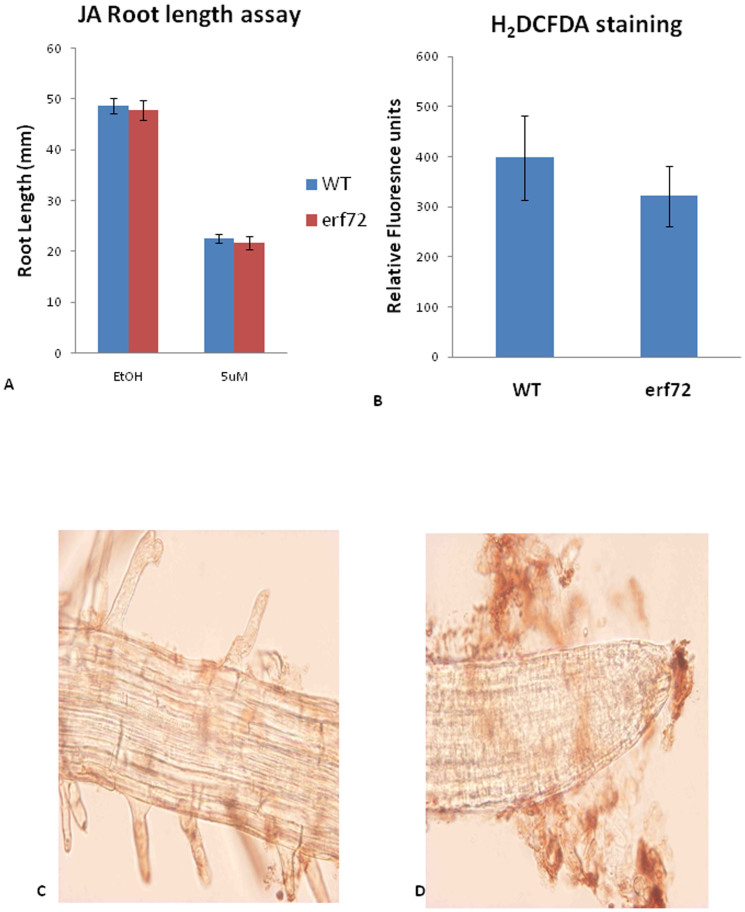
*erf72* mutant showed no difference on JA root inhibition & ROS measurement. (A) Bars represent mean root lengths ±SE of three independent replicates of 10 plants each. (B) Three biological replicates of 4 weeks-old plants were collected, and each biological replicate had 10 pooled plants. The Y-axis indicates the fluorescence reading under a plate reader. (C) Mock control and (D) *F. oxysporum*-infected roots by using DAB staining. In (D), it can be seen that fungus surrounds the outside area of the root.

As insensitivity to jasmonate has also been implicated in resistance to *F. oxysporum* we also quantified root growth of *erf72* mutants on MeJA-containing agar plates (Figure 4). These results showed no difference in root growth between the *erf72* mutant and WT. Therefore, with the exception of increased *CHI-B* expression, the *erf72* mutant appears un-affected in SA- and JA-associated defense gene expression or MeJA-mediated root inhibition. The heightened chitinase expression in *erf72* plants prior to infection may contribute towards its increased resistance against *F. oxysporum*.

*ERF72* has been shown to suppress programmed cell death in both plants and yeast when induced by the Bax protein, a pro-apoptotic protein from mammals^23^,^30^. In addition, over-expression of *ERF72* provided tobacco cell lines with increased tolerance to H_2_O_2_ treatment and led to up-regulation of the *PLANT DEFENSIN1.2* (*PDF1.2*) gene and *GLUTATHIONE S-TRANSFERASE6* (*GST6*) gene involved in plant defense and ROS responses^23^. We therefore hypothesized that the *erf72* mutant may have altered ROS responses and may be responsible for the change in *F. oxysporum* resistance in this mutant. We examined ROS content using 3, 3′-diaminobenzidine (DAB) staining of infected WT roots at 48 h post infection. However, examination of mock and *F. oxysporum*-infected roots showed no pathogen inducible ROS production using DAB staining (Figure 4). Therefore, a large ROS response is not produced in response to *F. oxysporum* infection in *Arabidopsis* roots. To examine whether there is a difference in H_2_O_2_ content in WT and *erf72* mutant plants, we quantified fluorescence after incubation of ground root tissue with 2′,7′-dichlorodihydrofluorescein diacetate (H_2_DCFDA). However, no difference could be detected between the roots of infected WT and *erf72* plants at 48 h post infection (Figure 4). Being a compatible interaction, it is perhaps not surprising that a large oxidative burst is not produced in *Arabidopsis* roots in response to *F. oxysporum* infection which appears to successfully circumvent PR gene expression and ROS production. However, it should be noted that subtle changes in ROS homeostasis may also provide resistance through enhanced defense signaling or through controling cell death pathways^31^. Therefore, more sensitive detection methods may be required to determine whether a T-DNA insertion in *erf72* leads to subtle changes in ROS signaling.

### The *pub22/23/24* triple mutant shows resistance to *F. oxysporum*

While predominantly studied in the leaves, the MAMP response has recently been shown to also be active in the roots of *Arabidopsis*^32^. Millet et al.^32^ used callose staining and GUS-promoter constructs to show that the MAMP response is inducible in roots by a range of elicitors. Similarly to the leaves, the root MAMP response can be effectively suppressed by the application of *P. syringae* or the jasmonoyl-isoleucine analog, coronatine, suggesting a possible role for JA in suppressing the root MAMP response^32^. In addition, the root colonizing fungus *Piriformospora indica* has recently been found to use JA signaling to suppress the MAMP response to support greater colonization^33^. As *F. oxysporum* is known to require JA signaling components to promote susceptibility and has been shown to induce JA-associated gene expression in the roots and shoots^17^,^18^; (Table 2), we hypothesized that *F. oxysporum* may also suppress MAMP responses via the JA pathway to allow greater infection.

**Figure 5:**
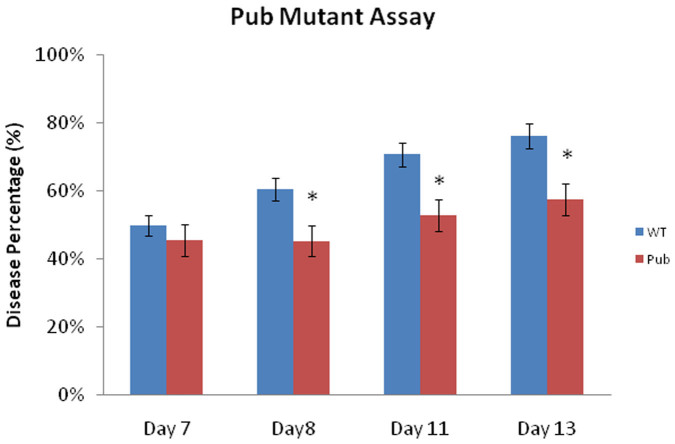
*Arabidopsis pub22/23/24* triple mutant plants showed increased resistance against *F. oxysporum*. The disease score represents the average proportion of symptomatic leaves per total leaves per plant. An asterisk (*) indicates significant differences (*p* < 0.05) according to Student's t test; bars represent mean values ±SE of three independent replicates of 10 pooled plants each. Pub-*pub22/23/24* triple mutant.

To explore whether an enhanced MAMP response could provide increased resistance to *F. oxysporum*, we inoculated the *pub22/pub23/pub24* triple mutant which lacks the PUB22, PUB23 and PUB24 U-box type E3 ubiquitin ligases. The *pub22/pub23/pub24* triple mutant has been shown to display increased resistance to *P. syringae* and the biotroph *Hyaloperonospora arabidopsidis* and also reduced colonization by *P. indica* due to a heightened MAMP response^33^,^34^. We inoculated the *pub22/pub23/pub24* mutant with *F. oxysporum* and found that the triple mutant also possessed increased resistance to *F. oxysporum* (Figure 5). This suggests that a heightened MAMP response may provide increased protection against *F. oxysporum* infection, but this process may be independent of ERF72.

## Discussion

In comparison to leaf-infecting pathogens there are relatively few studies of root pathogens due to the difficulty in observing the infection process in an unobtrusive manner. The exploration of the defense response in the leaves has provided vast insights into the main plant defense pathways that are activated in response to a pathogen attack. However, whether the defense pathways act in a similar manner in roots is still to be established.

*F. oxysporum* is a systemic pathogen that infects root tissue and travels to the root vasculature to cause disease in the stem and leaf tissue. Microarray analyses^15^,^17^ as well as a number of functional genomic type analyses^13^,^14^,^16^,^18^ have been performed to identify the signaling processes that are required for resistance against *F. oxysporum* in *Arabidopsis*. However these studies have primarily focussed on the leaf tissue and it is likely that the leaf may not be the ideal location for identifying resistance mechanisms against a root-infecting pathogen. Recently it has been shown that the JA co-receptor, COI1 is required for susceptibility to *F. oxysporum* and the *coi1* mutant shows almost complete resistance^18^,^19^. In addition, other mutants deficient in JA-associated gene expression pathways such as the *myc2* and *pft1* mutants are resistant to *F. oxysporum*^16^,^17^. These findings suggest that manipulation of the plant's JA signaling pathway is required for disease progression. Interestingly, grafting studies revealed that the resistance of *coi1* depended primarily on a *coi1* mutant rootstock suggesting that *coi1*-dependent resistance occurs in the roots^18^. To examine the resistance response in the roots in more detail, we profiled genome-wide gene expression of *F. oxysporum*-infected root samples collected at 48 h after infection. This time point was chosen to compare with a previously conducted microarray analysis performed on the leaf tissue.

In contrast to gene expression in the leaves, the roots of infected plants showed many more down-regulated genes as opposed to up-regulated genes. Also in contrast to the leaf array was the relative absence of defensin or pathogenesis-related (PR) protein gene expression in the infected root tissue. In infected roots, only one relatively uncharacterized defensin gene was activated but the expression was below the 1.5 fold cut-off used for selecting differentially expressed genes. Dowd et al.^35^ performed a microarray experiment on cotton infected with *F. oxysporum*. The authors found similar results with gene repression also being more predominant than induction in *F. oxysporum*-infected cotton roots. Dowd et al.^35^ also found little change in defense-related genes in the roots, however observed induced expression in the leaves, which is similar to the findings of our *Arabidopsis* leaf and root microarray analyses (Table 2)^17^. These observations possibly suggest that gene repression in the root tissue by *F. oxysporum* infection may contribute to the susceptibility of the infected plant.

The comparison of differentially expressed genes between our root analysis and the *F. oxysporum* leaf microarray showed only three genes in common between these two microarrays, suggesting that the gene expression changes in response to *F. oxysporum* infection are fundamentally different in the root and leaf tissue. Consistent with these findings, Attard et al.^36^ reported that the pattern of early defense mechanisms against *Phytophthora parasitica* clearly differs between roots and leaves in *Arabidopsis*. This appears to be an appropriate response for hemi-biotrophic pathogens such as *F. oxysporum* and *P. parasitica*, as the gene expression changes that occur in the roots, may be prioritized to perception of the pathogen and preventing penetration of the root tissue during the biotrophic stage, whereas the leaves may instead be acting to limit symptom development as a result of the switch to the necrotrophic stage. Similarly, Schlink^37^ found that gene expression changes were different in *Fagus sylvatica* in the early biotrophic stages compared to the later necrotic stages during *Phytophthora citricola* infection.

Although there was little overlap between the leaf and root differentially expressed genes in response to *F. oxysprorum*, comparisons with publically available microarray data showed that a subset of our root-induced genes were JA-responsive and therefore is somewhat similar to what was found for the leaf microarray where a proportion of the induced genes were JA-related^15^. The majority of the genes that were suppressed by *F. oxysporum* infection in *Arabidopsis* roots were also suppressed by FLG22 treatment, a peptide often used to analyse the plant MAMP response. This result suggests that *Arabidopsis* is able to recognize *F. oxysporum* and may switch off similar non-defensive pathways to co-ordinate a successful defense response. However, we could not find co-activation of FLG22-induced genes or other MAMP associated genes in our array experiment and it appears plausible that *F. oxysporum* may be suppressing genes associated with the root MAMP response as has been previously shown for *P. syringae* strain DC3000 and *P. indica* on *Arabidopsis* roots^32^,^33^. To test whether we could increase resistance by boosting the hosts MAMP response, we inoculated the *pub22/23/24* triple mutant with *F. oxysporum* and found the triple mutant to be more resistant comparative to the WT. The *PUB22/23/24* genes encode U-box type E3 ubiquitin ligases and act as negative regulators of MAMP-triggered immune responses^34^. Immune responses activated in the *pub22/23/24* mutant included the oxidative burst, map-kinase activity, and transcriptional activation of ROS and MAMP associated marker genes. The *pub* triple mutant has previously been shown to possess increased resistance to the hemi-biotroph *P. syringae*, the obligate oomycete, *H. arabidopsidis*, and also reduced colonization of the symbiotic fungus *P. indica*. Therefore, enhancing the MAMP response can increase resistance to a variety of organisms including *F. oxysporum*.

Millet et al.^32^ used callose staining and GUS-promoter constructs to show that the MAMP response is inducible in roots by a range of elicitors, and can be suppressed by the JA-Ile analog, coronatine. The suppression of the root MAMP response by coronatine required the JA co-receptor COI1 and the JA-associated transcription factor MYC2, but did not require suppression of the SA pathway^32^. Similarly we have previously shown that the *coi1* and *myc2* mutants are resistant to *F. oxysporum* and that the resistance observed in the *coi1* mutant does not require activation of the SA pathway^16^,^18^. Therefore these JA signaling components are required for both, susceptibility to *F. oxysporum* as well as suppression of the MAMP response. It is possible that the resistance phenotypes of *coi1* and *myc2* to *F. oxysporum* may be due to both, a reduced JA-dependent senescence in leaves and the ability of the fungus to suppress the MAMP response in these mutants.

Interestingly, Jacobs et al.^33^ indicated that the ability of *P. indica* to suppress host immunity is compromized in the jasmonate mutants *myc2* and *jasmonate resistant1-1* (*jar1-1*). Thus, JA signaling is also utilized by *P. indica* to suppress early root responses. In response to incompatible arbuscular mycorrhizae (AM), plants react with an increase in SA levels. However in compatible interactions, SA levels are reduced as the fungus colonizes the cortex, and then induction of JA biosynthesis occurs in arbuscule containing cells^38^. Therefore it is possible that *F. oxysporum* might hijack an ancestral pathway for microbial communication to evade the host defense response. In addition, different strains of *F. oxysporum* have previously been shown to produce a variety of JA compounds including JA-Ile^20^,^39^. This could suggest that *F. oxysporum* may use JA-ile and other JA compounds to suppress the MAMP response in order to gain entry to the plant root. Further investigation of the role of fungal-derived jasmonate in the root interaction with *F. oxysporum* is required to confirm these hypotheses.

Through selection of T-DNA insertion mutants of genes differentially expressed in infected roots, we were able to identify a role for the *ERF72* gene in susceptibility to *F. oxysporum*. *ERF72* encodes an AP2/ERF transcription factor that has been shown to suppress programmed cell death^23^. Expression of *ERF72* could suppress cell death in both plants and yeast when induced by the Bax protein, a pro-apoptotic protein from mammals^23^,^30^. Over-expression of *ERF72* in plants leads to up-regulation of the *PLANT DEFENSIN1.2* (*PDF1.2*) gene and *GLUTATHIONE S-TRANSFERASE6* (*GST6*) gene involved in plant defense and ROS signaling^23^. Our examination of the T-DNA insertion mutant showed no change in *PDF1.2* expression or in the expression of other JA- and SA-related defense genes. However it is likely that the WT-like expression of *PDF1.2* is due to redundancy of the other ERF transcription factors maintaining their expression. Interestingly, we found an increase in the expression of the *CHI-B* gene under mock conditions. Increased *CHI-B* expression may provide better protection against *F. oxysporum* within the roots. However, analysis of infection levels in the *erf72* mutant roots showed no significant difference in colonization between the WT and the mutant. Therefore the *erf72* mutant does not restrict the growth of the pathogen within the roots and reduced symptom development in the leaves of *erf72* is perhaps due to a greater tolerance of the pathogen within the root system, resulting in a suppressed symptom-causing defense response. Further investigation is needed to determine additional genes that may provide increased tolerance in *erf72*. We conclude that investigation of *F. oxysporum* responsive genes in the roots and characterising their roles in plant defense is a promising area to uncover the strategies used by root microbes to suppress host resistance, and this could provide useful tools to reduce losses in crop species to root-infecting plant pathogens that are often unaffected by pesticide treatments.

## Experimental procedures

### Plant growth and pathogen inoculation

*Arabidopsis thaliana* (Col-0) seeds were sown onto sterilized moist soil (UC mix) and incubated at 4°C in the dark for 3 days, to synchronize the germination of seeds. *Arabidopsis* seedlings were then grown in growth cabinets at 25°C, with an 8 h photoperiod (160 μE m^−2^s^−1^). After 2 weeks, seedlings were transferred to 30-well trays, and grown until the six to eight leaf stage. The *F. oxysporum* isolate used in this study was strain Fo5176 obtained from Dr. Roger Shivas, Queensland Plant Pathology Herbarium, Queensland Department of Agriculture, Fisheries and Forestry (DAFF), Brisbane, Australia. This strain was originally isolated from glasshouse-grown *Brassica oleracea* plants, and is highly virulent on *Arabidopsis* accessions, including Col-0. Sequence information for Fo5176 is available at Genbank under accession number AFQF00000000. Plants were inoculated with *F. oxysporum* as described previously^12^. Briefly, at 1 h after the start of the photoperiod (t = 0 h) the plants were gently uprooted and dipped for 15 s in fungal spore suspension with a concentration of 10^6^ spores/mL in water and then replanted. Mock plants were dipped in water and replanted. Root tissues were harvested at 48 h after inoculation (three independent biological replicates with pools of 40 plants each). Once the root samples were harvested, the root tissue was snap-frozen in liquid nitrogen. Additional experiments (three independent biological replicates with pools of 40 plants each) were carried out for the *F. oxysporum* qRT-PCR time course analyses. The *pub 22/23/24* triple mutant^34^ was kindly provided by Marco Trujillo. Statistical analyses were performed using Student's t-test using SPSS statistic version 20.0.0.

### Microarray analyses

RNA from *Arabidopsis* roots was extracted using the SV Total RNA Isolation System (Promega, USA). The RNA from mock- and *F. oxysporum*-inoculated samples was reverse-transcribed and labelled with Cy3 and Cy5 fluorescent dyes, respectively. The labelled cDNA samples were then hybridized onto 4 × 44 K Agilent *Arabidopsis* Gene Chip arrays (Agilent Technologies, USA). The labelling and hybridization steps were performed by the Australian Genome Research Facility (AGRF, Victoria, Australia). Signal intensities were extracted from scanned microarray images using Agilent Feature Extraction version 10.5.11 software (Agilent Technologies). The extracted data were analysed using Integromics Biomarker Discovery (Integromics Granada, Spain), and normalized within-arrays using the Loess algorithm, and between arrays using the Quantile normalization method.

Differentially expressed and statistically significant genes were selected based on the following cut-off criteria. The first criterion was that genes had to present fluorescence signals that were greater than background signal (gisPosAndSignif = 1 and risPosAndSignif = 1) by the Agilent Feature Extraction in both Cy3 and Cy5 channels. Secondly, the above genes with p-values < 0.05 using a parametric-based test (Welch T-test) were considered statistically significant. Finally, those genes that met the above listed criteria and presented a ratio (normalized red/normalized green) > 1.5 and < 0.68 were considered as up- and down- regulated genes, respectively.

### Real-time quantitative reverse transcriptase PCR (qRT-PCR) analyses

Total RNA from roots for qRT-PCR analyses were isolated using the SV Total RNA Isolation Kit (Promega). The concentration and quality of the RNA were measured with a spectrophotometer (NanoDrop® ND-1000) and a 1% agarose gel, respectively. cDNA synthesis was performed with 0.2 μg root RNA in 13.25 μL, using the SuperScript™ III RT kit (Invitrogen) as follows. A total of 0.2 μL of 100 μM oligo-dT, 0.05 μL of 3 μg/μL random hexamers (Invitrogen) and 1 μL of 10 mM dNTPs were added to a final volume of 20 μL. The mixture was denatured at 65°C for 5 min followed by 2 min of chilling on ice. A total of 4 μL of 5× first strand buffer, 1 μL of 0.1 mM DTT (Invitrogen) and 0.5 μL (200 U/μL) SuperScript^TM^ III Reverse Transcriptase were added, followed by incubation at 52°C for 50 min and 70°C for 15 min. The resulting cDNA was subsequently diluted to a final concentration of 20 ng/μL of input RNA for qRT-PCR.

Gene expression analysis by qRT-PCR was carried out in 384-well plates using an ABI PRISM 7900HT Sequence Detection System (Applied Biosystems). Each reaction contained 5 μL of SYBR green and 2 μL of 200 nM of each gene-specific primer pair and 20 ng/μL of cDNA template to a final volume of 10 μL. The PCR primer efficiency (E) of each primer pair in each individual reaction was calculated from the changes in fluorescence values (ΔRn) of each amplification plot, using LinReg PCR software^40^. E values for each gene were averaged across all samples, except in cases where linear regression of amplification plots yielded a *R^2^* value of less than 0.99, in which case the derived E value for that sample was omitted from the calculation of mean E value. Amplification plots were analysed using a threshold of 0.20 to give a cycle threshold (Ct) value for each gene and cDNA combination. Gene expression levels relative to the *Arabidopsis* housekeeping genes *β-ACTIN 2* (AT3G18780), *β-ACTIN 3* (AT3G53750) and *β-ACTIN 7* (AT1G49240) were calculated for each cDNA sample using the following equation: The gene transcript levels relative to actin = (E gene^∧^(-Ct gene))/(E *Actin*^∧^ (-Ct *Actin*)). The qRT-PCR experiments were analysed using Student's *t*-test.

### *F. oxysporum* GUS histochemical assay

The *uidA*(GUS)-expressing *F. oxysporum* transgenic strain was obtained from Dr. Ming Bo Wang's laboratory at CSIRO. The plants were inoculated with *F. oxysporum* GUS spores at a concentration of 1 million spores/mL. The plant root tissue was cleared with 100% ethanol after 14 d post inoculation. The roots were incubated at 37°C in staining solution overnight. The staining solution contained 2 mM X-Gluc (5-bromo-4-chloro-3-inoyl β-D-glucuronide cyclohexylammonium salt in dimethyl formamide), 0.1% Triton, 0.5 mM K_3_Fe(CN)_6_, 0.5 mM K_4_Fe(CN)_6_.3H_2_O, 10 mM EDTA and 50 mM K or (Na)PO_4_ buffer pH7.0. Following X-Gluc incubation, the root tissue was de-stained with 100% ethanol for 5 min and cleaned with fresh sterilized water. The tissue slide was observed under a compound microscope (Olympus).

### H_2_O_2_ quantification

Hydrogen peroxide quantity was measured according to the method of Joo et al.^41^. A total of 30 mg of liquid nitrogen-ground plant sample was extracted in 1 mL Tris-HCl buffer (10 mM, pH7.3). The homogenate was centrifuged at 13,000 xg for 5 min at 4°C. The supernatant was taken and centrifuged again under the same conditions. H_2_O_2_ was detected using the dye 2′,7′-dichlorofluorescein diacetate (H_2_DCFDA). This indicator is a cell permeable non- fluorescent probe, but it switches to high fluorescence during oxidation. The assay mixture contained 20 μM H_2_DCFDA final concentration (a stock of 100 mM in DMSO was prepared) and 100 μL extract. The volume was prepared to 250 μL with 10 mM Tris-HCl buffer (pH7.3). In parallel with each sample, catalase (300 unit/mL, Sigma) was added to subtract with dye. The fluorescence was measured at 40 min after H_2_DCFDA staining using a fluorometer (Fluoroskan Ascent).

### DAB Staining

Detection of hydrogen peroxide was conducted using 3,3′-diaminobenzidine (DAB) from Sigma-Aldrich. Briefly, plants were inoculated with *F. oxysproum* suspension and the root tissues were collected and mixed 1 mL of DAB liquid buffer solution with 30 μL of DAB liquid chromogen. After staining, the plant tissues were rinsed with distilled water for 5 times and then observed under a microscope (Olympus BX60F5).

## Author Contributions

Y.C., B.K. and P.S. wrote the main manuscript text, and C.W. and I.V. performed Microarray experiment. F.M. normalised the Microarray data. Y.C. did all other experiments. Y.C., B.K. and P.S. reviewed the manuscript.

## Supplementary Material

Supplementary Information SUPPLEMENTARY Figures

## Figures and Tables

**Table 1:** Genes that were significantly down-regulated greater than two-fold by *F. oxysporum* infection in *Arabidopsis* root tissue

TAIR ID	Gene Description	Fold Change	T-test
AT5G17220	*GLUTATHIONE S-TRANSFERASE 12* (*GST12*)	0.24	0.02
AT3G22120	Cell wall-plasma membrane linker protein homolog	0.25	0.021
AT1G29930	*CHLOROPHYLL A/B BINDING PROTEIN 1* (*CAB1*)	0.28	0.048
AT5G48485	*DEFECTIVE IN INDUCED RESISTANCE 1* (*DIR1*)	0.30	0.029
AT1G60590	Pectin lyase-like protein	0.31	0.015
AT5G25980	*THIOGLUCOSIDE GLUCOHYDROLASE 2* (*TGG2*)	0.36	0.004
AT3G16770	*ETHYLENE RESPONSE FACTOR 72* (*ERF72*)	0.40	0.017
AT3G50440	*METHYL ESTERASE 10* (*MES10*)	0.40	0.003
AT5G26000	*THIOGLUCOSIDE GLUCOHYDROLASE 1* (*TGG1*)	0.40	0.042
AT5G13930	*CHALCONE SYNTHASE* (*CHS*)	0.40	0.020
AT5G46110	*ACCLIMATION OF PHOTOSYNTHESIS TO ENVIRONMENT 2*(*APE2*)	0.42	0.030
AT1G61190	Response to auxin stimulus	0.44	0.013
AT3G26650	*GLYCERALDEHYDE 3-PHOSPHATE DEHYDROGENASE A SUBUNIT* (*GAPA*)	0.48	0.045
AT1G29490	SAUR-like auxin-responsive protein	0.48	0.034
AT5G62630	HIPL2 protein precursor	0.49	0.020

**Table 2:** Genes that were significantly up-regulated greater than 1.5-fold by *F. oxysporum* infection in *Arabidopsis* roots

TAIR ID	Gene Description	Fold Change (infected/mock)	T-test
AT3G55970	*JASMONATE-REGULATED GENE 21* (*JRG21*)	2.66	0.005
AT4G22610	Lipid transport protein	2.14	0.024
AT1G30135	*JASMONATE ZIM DOMAIN PROTEIN 8* (*JAZ8*)	1.58	0.030
AT3G62760	*GLUTATHIONE S–TRANSFERASE 13* (*GST13*)	1.55	0.007
AT2G38240	Oxidoreductase	1.79	0.009
AT5G19110	Eukaryotic aspartyl protease protein	1.73	0.018
AT2G26370	ML domain-containing protein	1.70	0.027
AT1G61080	Proline-rich family protein	1.66	0.009
AT3G44870	S-adenosyl-L-methionine-dependent methyltransferase	1.66	0.005
AT3G30740	Ribosomal Protein	1.59	0.032
AT1G04270	*CYTOSOLIC RIBOSOMAL PROTEIN S15* (*RPS15*)	1.58	0.019
AT1G21528	unknown protein	1.56	0.005
AT1G13510	unknown protein	1.55	0.001
AT2G36080	DNA-binding protein	1.55	0.010
AT5G26070	Hydroxyproline-rich glycoprotein	1.54	0.009
AT2G27710	60S acidic ribosomal protein P2	1.54	0.006
AT2G39460	RNA binding/structural constituent of ribosome	1.50	0.024
