# Supplementary material for: Root defense analysis against Fusarium oxysporum reveals new regulators to confer resistance
Source: Sci Rep. 2014 Jul 7;4:5584. doi: 10.1038/srep05584 (PMC4083284; doi:10.1038/srep05584)
Supplement: Supplementary Information — SUPPLEMENTARY Figures [file srep05584-s1.pdf]

Root defense analyses against *Fusarium oxysporum* reveals new regulators to confer resistance

Yi Chung Chen, Chin Lin Wong, Frederico Muzzi, Ido Vlaardingerbroek, Brendan N. Kidd, Peer M. Schenk*

Plant-Microbe Interactions Laboratory, School of Agriculture and Food Sciences, The University of Queensland, Brisbane, Queensland 4072, Australia

*Corresponding author email: p.schenk@uq.edu.au

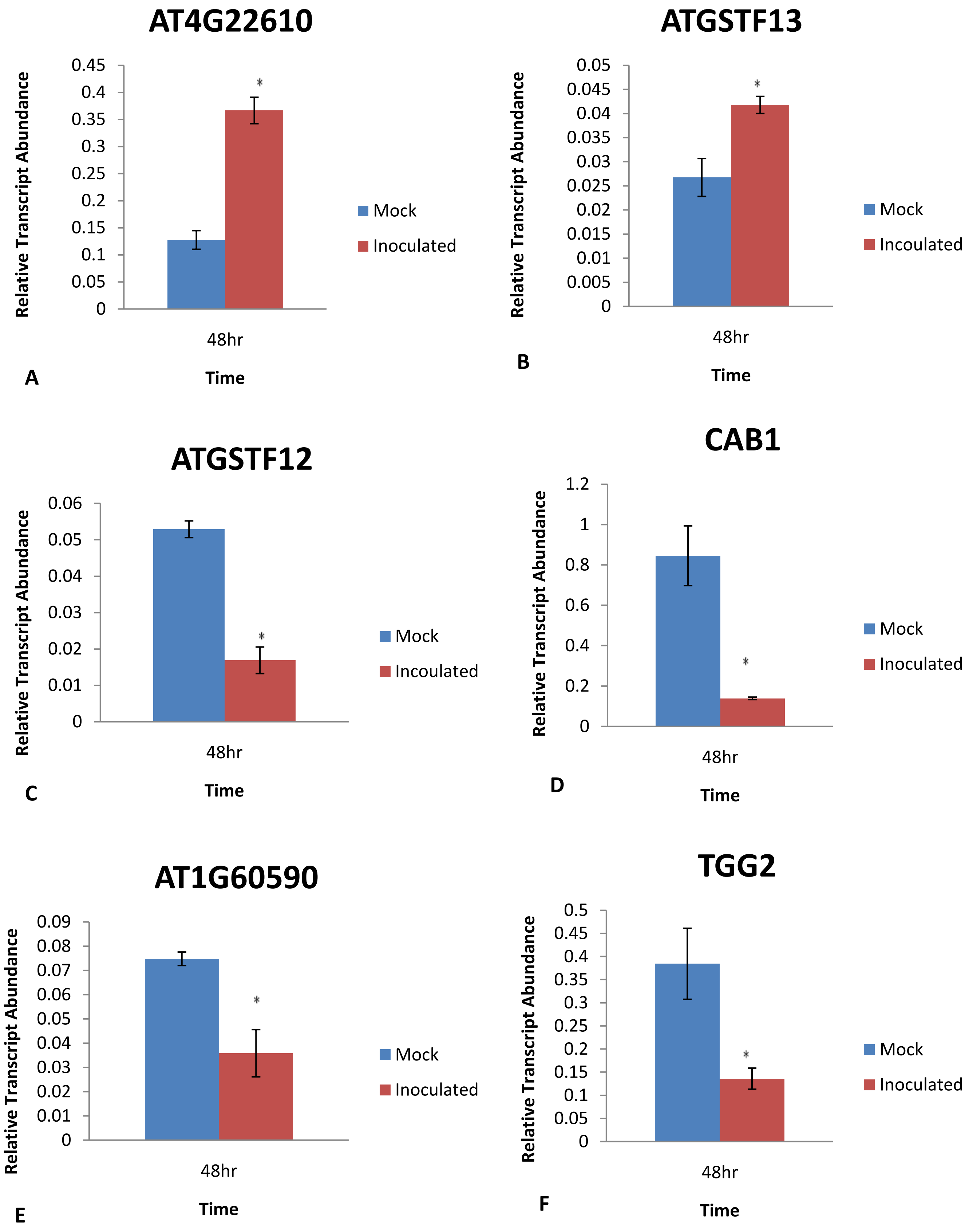

Supplementary Figure 1 (A-F). Differential gene expression in *Arabidopsis* roots following infection by *Fusarium oxysporum* at 48 h post inoculation compared to mock treatment. Shown are qRT-PCR data from three independent biological replicates, each containing a pool of 40 plants.

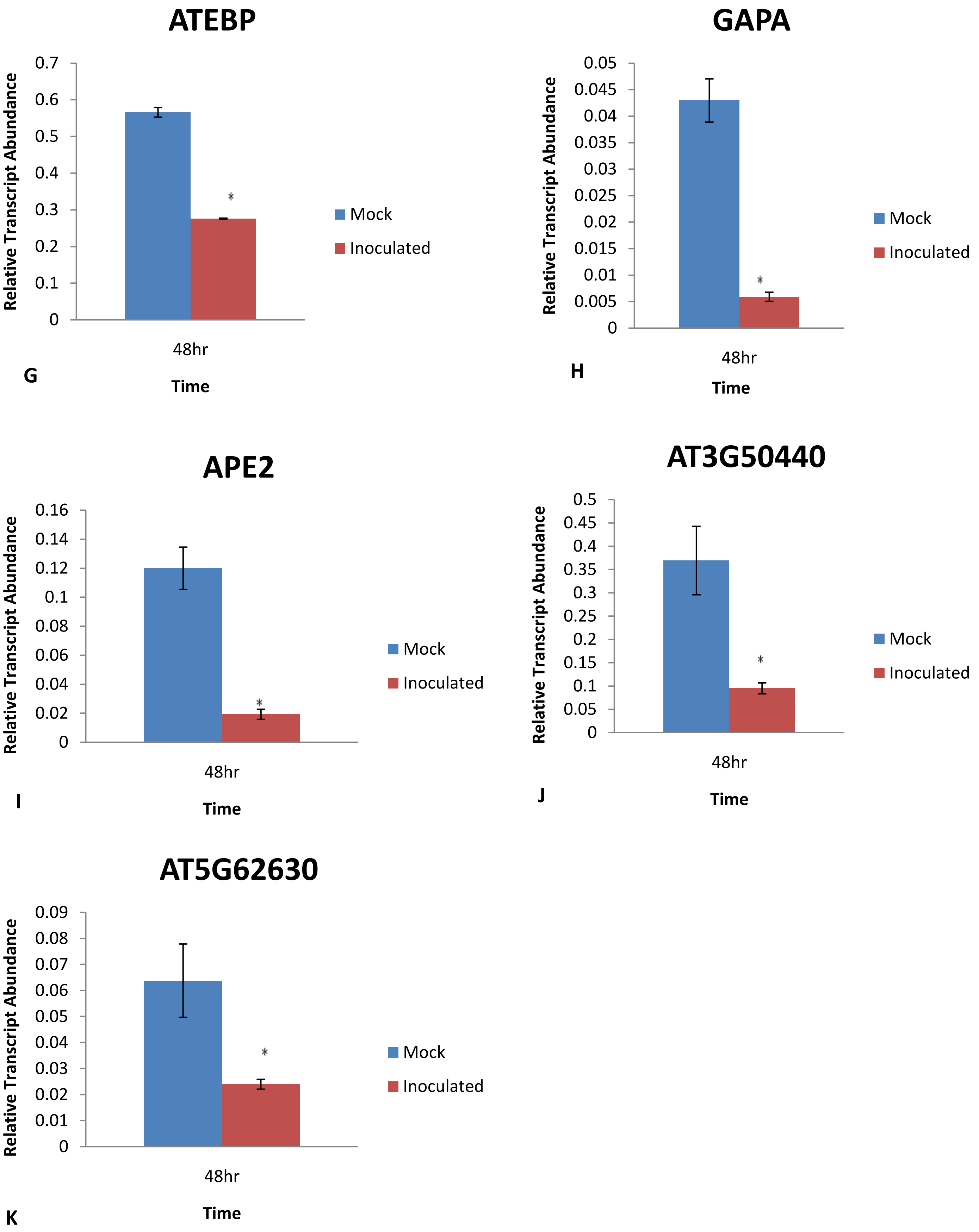

Supplementary Figure 1 (G-K). Differential gene expression in *Arabidopsis* roots following infection by *Fusarium oxysporum* at 48 h post inoculation compared to mock treatment. Shown are qRT-PCR data from three independent biological replicates, each containing a pool of 40 plants.

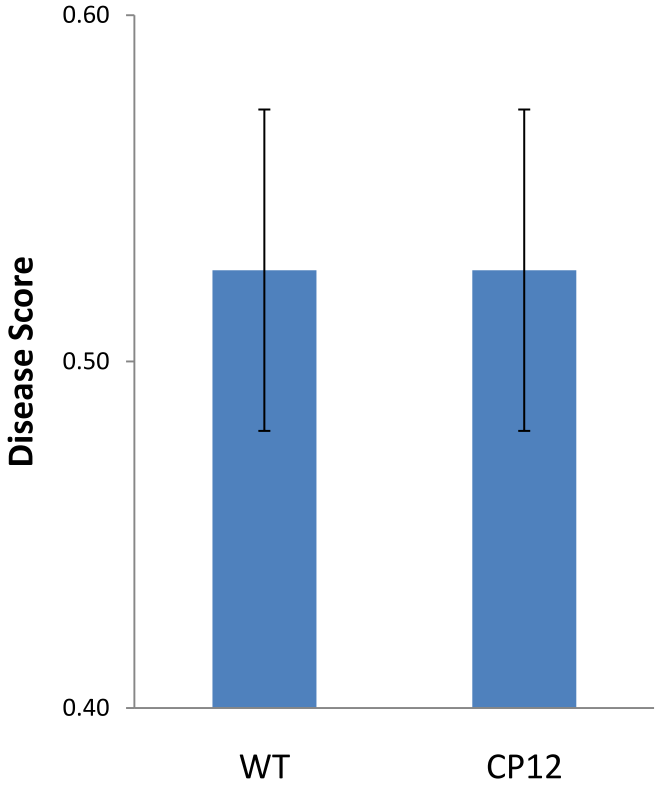

Supplementary Figure 2. Disease score for *cp12* mutant. The disease score represent the average proportion of symptomatic leaves per total leaves per plant. Bars represent mean values ±SE of three independent replicates of 10 pooled plants each.

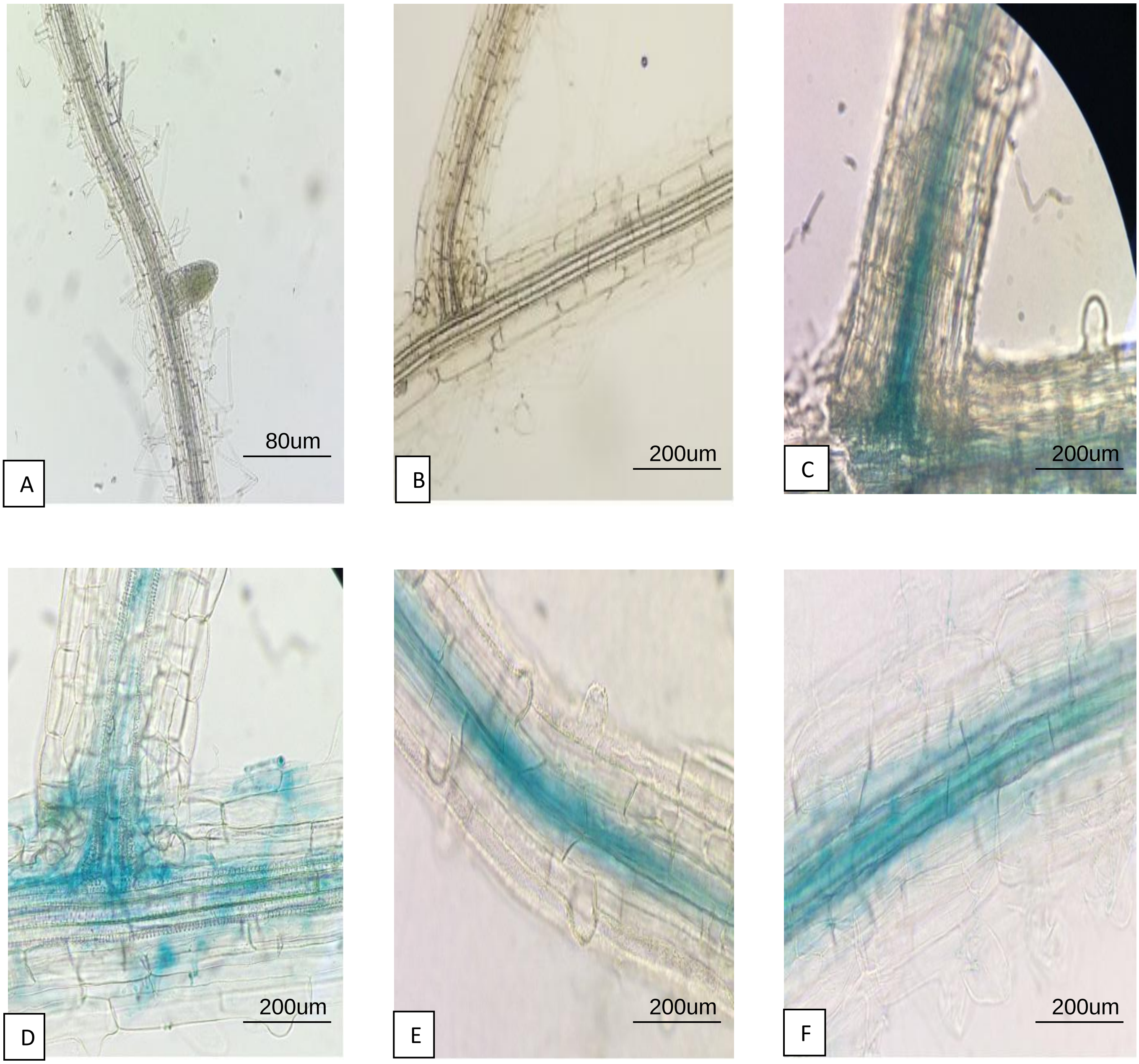

Supplementary Figure 3. GUS expression of *F. oxysporum* hyphae colonizing WT & *erf72* *Arabidopsis* plants. (A) Mock control of WT (Col-0) plant root (B) Mock control of *erf72* root (C,E) *F. oxyspoorum* expression on WT (Col-0) plant root (D, F) *F. oxysporum* expression on *erf72* root

Supplementary Table 1. *Arabidopsis thaliana* genes found to be significantly repressed more than 1.5 fold but less than 2 fold in *F. oxysporum*-infected roots compared to mock-inoculated roots.

Common Name	Unique ID	Avg. Norm A	Avg. Norm M	Anti-Log2	T-test
ATGLR1.4	AT3G07520	10.90997409	-0.992360186	0.50279171	5.10E-04
PIF4	AT2G43010	10.21801438	-0.969815465	0.51082637	1.85E-03
CP12-1	AT2G47400	12.53256093	-0.971965298	0.51700732	3.42E-03
THI1	AT5G54770	12.89884341	-0.939209136	0.53200985	4.25E-02
CAT3	AT1G20620	15.49393219	-0.904881022	0.53343576	4.60E-02
AT4G00780	AT4G00780	11.51466893	-0.883622123	0.54372252	1.73E-02
PETC	AT4G03280	10.66825751	-0.883008036	0.54434555	2.38E-02
AT1G03495	AT1G03495	9.496486977	-0.743745497	0.54998874	4.92E-02
LSH1	AT5G28490	11.86556314	-0.872726376	0.5502214	6.17E-03
AT3G45160	AT3G45160	14.51579788	-0.913964297	0.55135116	2.76E-02
AT4G12470	AT4G12470	10.03300689	-0.852410503	0.55155468	2.67E-02
KNAT1	AT4G08150	10.91861785	-0.828291112	0.56581149	1.79E-02
ATSEH	AT2G26740	10.58346439	-0.809967833	0.56985825	1.07E-02
AT4G07742	AT4G07742	9.557648475	-0.767509114	0.5698831	7.88E-03
AT2G26500	AT2G26500	10.49190973	-0.802574519	0.57330504	2.52E-02
CYP71B18	AT3G26165	11.53007984	-0.802929824	0.5750255	8.29E-03
AT1G16260	AT1G16260	10.69933918	-0.786112063	0.58119209	1.40E-02
AT1G67860	AT1G67860	13.27946538	-0.805542531	0.5813031	3.69E-02
STM	AT1G62360	9.194569673	-0.735234507	0.5898456	6.24E-03
AT5G53820	AT5G53820	12.39010203	-0.758351983	0.59708255	7.29E-03
CYP96A12	AT4G39510	10.15925678	-0.736005573	0.59794424	2.39E-02
AT1G52220	AT1G52220	10.10916329	-0.741388883	0.598628	4.15E-02
ACD32.1	AT1G06460	11.19655375	-0.729134803	0.60424372	1.93E-02
AT5G19140	AT5G19140	11.41074788	-0.724714086	0.60590691	4.08E-02
CYP706A1	AT4G22690	12.33643292	-0.739199264	0.60609912	2.40E-02
AT3G23290	AT3G23290	10.89046414	-0.714788319	0.6071095	2.61E-02
AT5G62350	AT5G62350	15.71685427	-0.68522096	0.60860915	1.54E-03
AT3G18080	AT3G18080	12.48067394	-0.728013468	0.60997247	1.31E-02
PDC2	AT5G54960	13.3931817	-0.746226418	0.61135899	2.91E-02
AT3G18080	AT3G18080	12.56235442	-0.720302704	0.61439658	2.27E-02
COR15A	AT2G42540	10.20752672	-0.723098148	0.61591081	4.32E-02
AT1G45191	AT1G45191	9.511259815	-0.639283614	0.61688749	3.39E-02
ATLFNR1	AT5G66190	10.56278075	-0.694105891	0.61834072	4.37E-02
TT7	AT5G07990	9.806462436	-0.694487065	0.6183493	1.22E-02
AT5G50510	AT5G50510	10.192071	-0.684085699	0.62177479	2.03E-02
TIR	AT1G72930	10.94509293	-0.689907215	0.62258296	4.37E-02
AT5G19940	AT5G19940	11.68141518	-0.688740715	0.62427692	4.60E-02
AT1G19960	AT1G19960	12.17855794	-0.690124895	0.62468577	4.80E-02
AT3G54260	AT3G54260	14.55629356	-0.74019079	0.62728213	2.33E-02
AT5G59305	AT5G59305	9.467364599	-0.660384294	0.62942852	2.86E-02
AT3G61490	AT3G61490	12.2275336	-0.68123955	0.62981052	2.72E-02
FED A	AT1G60950	12.82728076	-0.683352142	0.63251798	3.96E-02
AT4G22485	AT4G22485	11.53699906	-0.662438708	0.63418704	1.34E-02
ANAC036	AT2G17040	10.5991472	-0.651908295	0.64165407	3.81E-02
AT1G67865	AT1G67865	11.92794348	-0.646129469	0.64362813	2.28E-02
AT1G48300	AT1G48300	12.94556129	-0.655765098	0.64415755	5.36E-03
PAP2	AT4G29080	12.54534803	-0.649991462	0.64419446	2.87E-02
ETR2	AT3G23150	11.17215535	-0.639792324	0.64531372	4.08E-02
AT5G09530	AT5G09530	14.87088136	-0.698956032	0.64587358	2.81E-02
PAP1	AT1G56650	9.585163256	-0.568518217	0.64656728	1.54E-02
AT5G02160	AT5G02160	10.6269218	-0.639530775	0.64726659	3.21E-02
AT1G14890	AT1G14890	15.08703144	-0.687626188	0.64990715	3.27E-02
AT4G37550	AT4G37550	10.61838005	-0.620960947	0.65215046	1.73E-03
FDH	AT5G14780	12.82871652	-0.641930561	0.6535428	4.85E-02
AT5G64110	AT5G64110	10.42940848	-0.602543596	0.65694209	2.97E-02
AT1G54410	AT1G54410	15.77101126	-0.527071693	0.65737292	2.42E-06
AT1G19530	AT1G19530	13.04807484	-0.630189879	0.66034258	3.64E-02

SUPPLEMENTARY FIGURE LEGENDS

Supplementary Figure 1. Differential gene expression in *Arabidopsis* roots following infection by *Fusarium oxysporum* at 48 h post inoculation compared to mock treatment. Shown are qRT-PCR data from three independent biological replicates, each containing a pool of 40 plants.

Supplementary Figure 2. Disease score for *cp12* mutant. The disease score represent the average proportion of symptomatic leaves per total leaves per plant. Bars represent mean values ±SE of three independent replicates of 10 pooled plants each.

Supplementary Figure 3. GUS expression of *F. oxysporum* hyphae colonizing WT & *erf72 Arabidopsis* plants. (A) Mock control of WT (Col-0) plant root (B) Mock control of *erf72* root (C,E) *F. oxyspoorum* expression on WT (Col-0) plant root (D, F) *F. oxysporum* expression on *erf72* root

Supplementary Table 1. *Arabidopsis thaliana* genes found to be significantly repressed more than 1.5 fold but less than 2 fold in *F. oxysporum*-infected roots compared to mock-inoculated roots.
